# Intracardiac tuberculomas caused by *Mycobacterium tuberculosis* in a dog

**DOI:** 10.1186/s12917-016-0731-7

**Published:** 2016-06-14

**Authors:** Olga Szaluś-Jordanow, Ewa Augustynowicz-Kopeć, Michał Czopowicz, Arkadiusz Olkowski, Andrzej Łobaczewski, Magdalena Rzewuska, Rafał Sapierzyński, Elżbieta Wiatr, Magdalena Garncarz, Tadeusz Frymus

**Affiliations:** Department of Small Animal Diseases with Clinic, Division of Infectious Diseases, Warsaw University of Life Sciences-SGGW, Nowoursynowska 159c Street, 02-776 Warsaw, Poland; Department of Microbiology, Institute of Tuberculosis and Lung Diseases, Płocka 26 Street, 01-138 Warsaw, Poland; Laboratory of Veterinary Epidemiology and Economics, Warsaw University of Life Sciences-SGGW, Nowoursynowska 159 Street, 02-776 Warsaw, Poland; Veterinary Clinic “Auxilium” Arkadiusz Olkowski, Królewska St. 64, 05-822 Milanówek, Poland; Department of Preclinical Sciences, Division of Microbiology, Warsaw University of Life Sciences-SGGW, Ciszewskiego 8 Street, 02-786 Warsaw, Poland; Department of Pathology and Veterinary Diagnostics, Warsaw University of Life Sciences-SGGW, Nowoursynowska 159 Street, 02-776 Warsaw, Poland; Third Department of Lung Diseases, Institute of Tuberculosis and Lung Diseases, Płocka 26 Street, 01-138 Warsaw, Poland

**Keywords:** Mycobacterium tuberculosis, Tuberculoma, Dog

## Abstract

**Background:**

This paper presents an unusual form of disseminated *Mycobacterium tuberculosis* infection in a dog. The infection lasted at least one year and its main gross lesions were massive cardiac tuberculomas. To the best of our knowledge, this is the first report of heart tuberculomas in a dog.

**Case presentation:**

A 9-year-old mixed-breed male dog weighing 10 kg was referred to the clinic for cardiological evaluation before general anesthesia. The echocardiography revealed a lump of about 20 mm in diameter in the area of the left atrium. Almost one year later the same dog was presented again in severe clinical state (fever, anorexia, weight loss, depression, cough, dyspnea, lymphadenomegaly, vomiting, recent episodes of fainting). Due to progression of the disease and poor effects of treatment the owner decided to euthanize the dog. Most prominent lesions observed during autopsy were diffuse pneumonia, fibrinous pericarditis and epicarditis as well as large, yellow, semisolid masses of caseous necrosis in the left and right atrium (30 mm and 15 mm in diameter, respectively). From both pulmonary and cardiac lesions *M. tuberculosis* was isolated on Lowenstein-Jensen slants and in Bactec *Mycobacteria* Growth Indicator Tube 960 liquid media, and confirmed by BD ProbeTec ET Direct Detection Assay and spoligotyping.

**Conclusion:**

Companion animals may occasionally suffer from tuberculosis but majority of cases probably remain misdiagnosed or undetected. Typically tuberculosis in dogs affects lungs and their regional lymph nodes. Even in humans tuberculomas are rare manifestation of mycobacterial infection, mostly seen in the central nervous system. Atypical location of main tuberculous lesions may account for lack of correct ante mortem diagnosis in this case.

## Background

Among several closely related species causing tuberculosis (TB) in humans and animals grouped in the *Mycobacterium tuberculosis complex* (MTC), the most important are *M. tuberculosis* and *M. bovis*. Both agents can infect and induce disease in several animal species, occasionally also in dogs [1, 2]. Typically, canine TB is a chronic disease affecting lungs and regional lymph nodes, however, lesions have been described also in many other organs [2]. In contrast to most other bacterial diseases a prolonged contact with an infected animal or human is required to transmit TB to dogs.

Historically, *M. bovis* infection was more common in dogs as a result of consumption of raw milk and contacts with infected cattle. This has changed along with eradication of bovine TB [2]. However, in some countries, e.g. the UK the incidence of TB in cattle has increased over the last 25 years [3]. Poland is officially free of bovine tuberculosis since 2009 [4].

As mentioned above, dogs may also occasionally suffer from tuberculosis caused by *M. tuberculosis*, for which a human considered the only reservoir host [2]. Though a significant progress has been made toward the elimination of tuberculosis from humans, this disease remains an important global problem, especially in developing countries. In 2014, an estimated 9.6 million people developed tuberculosis and 1.5 million died from the disease [5]. About 80 % these cases occur in South-East Asia and Western Pacific regions as well as in Africa. In Europe, the epidemiological situation of tuberculosis is much better and still improves. Nevertheless, 64 844 human cases were reported in 30 European Union/European Economic Area countries in 2013, what makes a notification rate of 12.7 per 100 000 population, with 77.9 % of them being newly diagnosed cases [6]. In Poland, the incidence of human tuberculosis has decreased more than 10-fold over last 50 years. For instance, in 1957 over 82 000 new cases were registered, while in 2013 only 6 403. The incidence in 2013 was 18.8 cases per 100 000 people, 88.3 % of them being newly recognized [7].

Though epidemiological data on canine TB are generally lacking [2] in many well-developed countries including Poland TB in humans and animals is considered an extremely rare condition and it is seldom numbered among differential diagnoses by veterinarians even if clinical presentation should raise suspicion of this disease. Therefore, TB cases tend to remain misdiagnosed or undetected and medical practitioners are at risk of exposure of pre-multidrug-resistant tuberculosis [8–10]. This report describes an unusual form of *M. tuberculosis* infection in a dog where massive intracardiac tuberculomas were the main manifestation.

## Case presentation

A 9-year-old mixed-breed male dog weighing 10 kg was referred to the clinic for echocardiography to exclude heart disease before surgery in general anesthesia due to dental sanation. According to the history, the dog had been healthy since being adopted at the age of about 4 months, and lived the whole time in this household. Though no clinical signs were visible, echocardiography revealed a nodule of about 20 mm in the area of the left atrium. A neoplasm was suspected, but the owner refused further diagnostic procedures.

Three months later the dog was referred to the clinic again because of depression, anorexia, weight loss, severe cough and rapid breathing. Clinical examination revealed severe dyspnea and enlarged abdomen indicating ascites. During ultrasound examination, large amount of free peritoneal fluid was confirmed, and massive pericardial effusion with cardiac tamponade was revealed. By pericardiocentesis about 100 ml of a yellow fluid was evacuated. Cytological examination of this fluid revealed an increased number of inflammatory cells, mostly neutrophils with nuclear degeneration. The material was sent for bacteriological examination and treatment with furosemide (3 mg/kg body weight twice daily) and cephalexin (10 mg/kg body weight twice daily) was initiated. Although from the pericardial fluid no bacterial growth was obtained on routine media, this therapy was continued for 9 days. The general and circulatory state of the patient improved quickly, and as early as on the 3rd day after pericadiocentesis echocardiography could be performed. This examination revealed a nodule 20x30mm in diameter located close to the left atrium, slight pericardial effusion, a thickened pericardial sac and mitral regurgitation. Measurement in the right parasternal short axis view were as follows: LA 1.71 cm, Ao 1.62 cm, LA/Ao 1.05, RV 0.74 cm, IVSs 0.88 cm, IVSd 0.74 cm, LVIDs 1.21 cm, LVIDd 2.50 cm, LVPWs 1.42, LVPWd 0.88 cm, FS 51 %, EF 84 %, LVOT 0.84 m/s and in the left parasternal view RVOT 0.77 m/s. As the general state of the patient was much better, the owner declined further diagnostics, but presented the dog on the 20th day post pericadiocentesis for a control examination, which revealed a small amount of fluid in the pericardium.

**Fig. 1:**
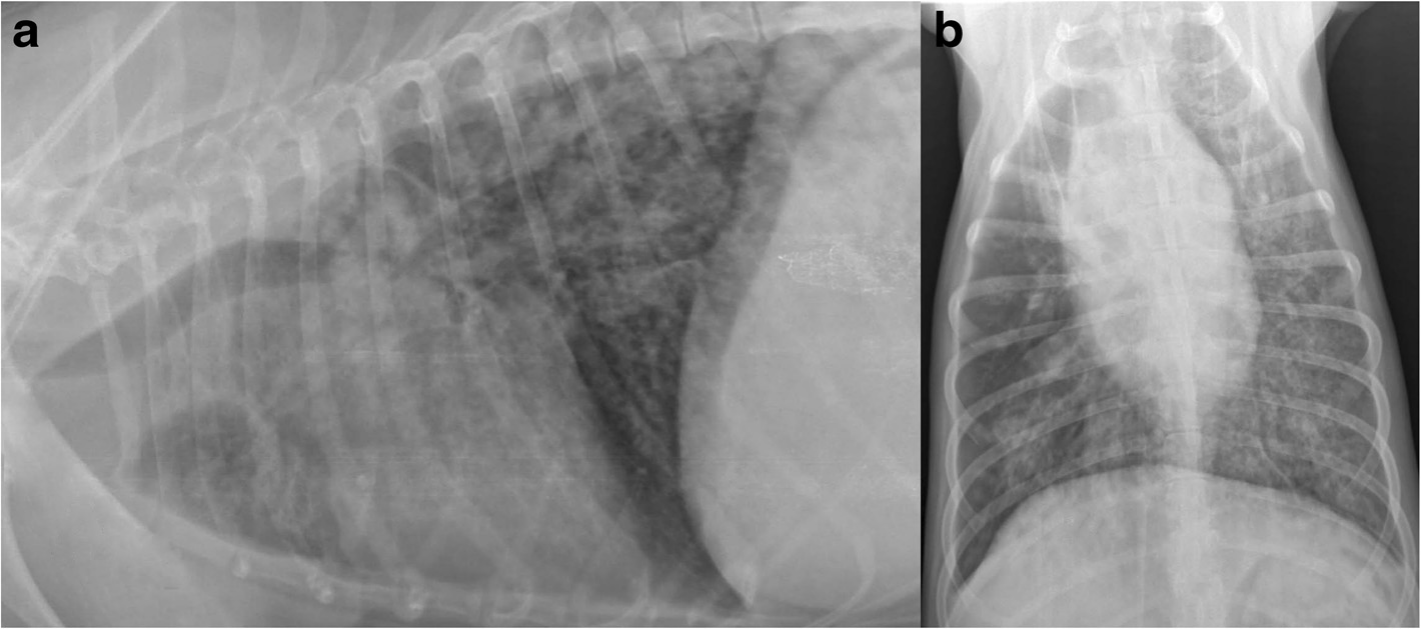
Thoracic radiographs from a dog. **a**) lateral, **b**) ventrodorsal

Almost one year after the first visit the patient was presented again with significant clinical worsening (fever, anorexia, weight loss, depression, cough, dyspnea, severe weakness, syncope). The abdominal ultrasonography revealed lesions indicating chronic disease of the liver (enlargement, parenchymal inhomogeneity and nodularity, degenerative signs, numerous hiperechogenic calcification-like foci about 1 mm in diameter, thickening of the gallbladder wall), spleen (degenerative remodeling of the parenchyma), kidneys (highly hyperechogenic foci, significant reduction in cortico-medullary differentiation, areas of fibrosis and mineralization, irregular and thickened capsules), pancreas (enlarged, hyperechogenic, heterogenic parenchyma, hyperechogenic peripancreatic adipose tissue), stomach (tchickened wall with post-infalmmatory remodeling), omentum (hyperechogenic), prostate (benign hypertrophy), urinary bladder (thickened wall, locally with significant reduction in layers differentiation, urine sediment and calculi up to 4 mm in diameter). Dorsoventral and right lateral thoracic radiographs (Fig. 1a, b) revealed bronchial changes in the whole left lung lobes and in the right caudal lobe, as well as unstructured interstitial lung patterns. A localized lung consolidation was also noted in the right middle lung lobe. Additionally, tracheobronchial lymphadenoomegaly has been revealed in the right lateral projection. During this visit the owner reported that almost 6 months earlier an oral therapy with amoxicillin and clavulanic acid had been performed by a private veterinarian because of voice change and nighttime cough of the dog, and about one month later another veterinarian had applied tylosin and diet change due to fever and diarrhea. Due to the progression of the disease, and neoplasia suspect, the owner refused further diagnostics and decided to euthanize the dog a few days later.

**Fig. 2:**
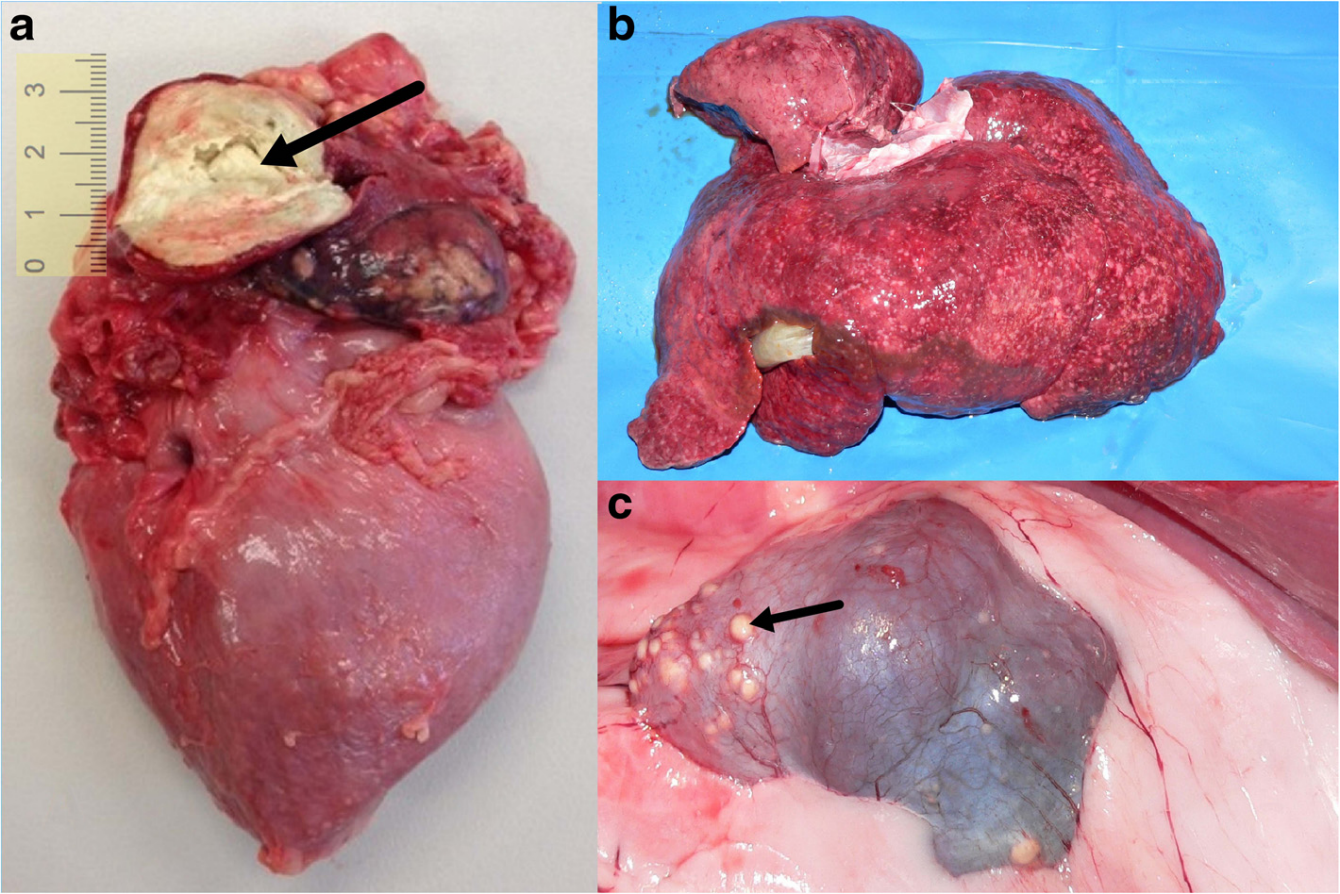
Autopsy appearance of altered internal organs of the dog affected with tuberculosis: **a**) tuberculoma in the left atrium (black arrow); **b** numerous small foci of granulomatous inflammation diffusely scattered thorough the liver; **c**) numerous subcapsular small foci of granulomatous inflammation in the kidney (black arrow), note irregular shape of the organ

Most prominent lesions seen during autopsy were fibrinous pericarditis and epicarditis, two large, yellow, semisolid masses of caseous necrosis in the left and right atrium (30 mm and 15 mm in diameter, respectively) (Fig. 2a), diffuse pneumonia, hepatomegaly (Fig. 2b), numerous subcapsular small nodules in the kidneys (Fig. 2c).

**Fig. 3:**
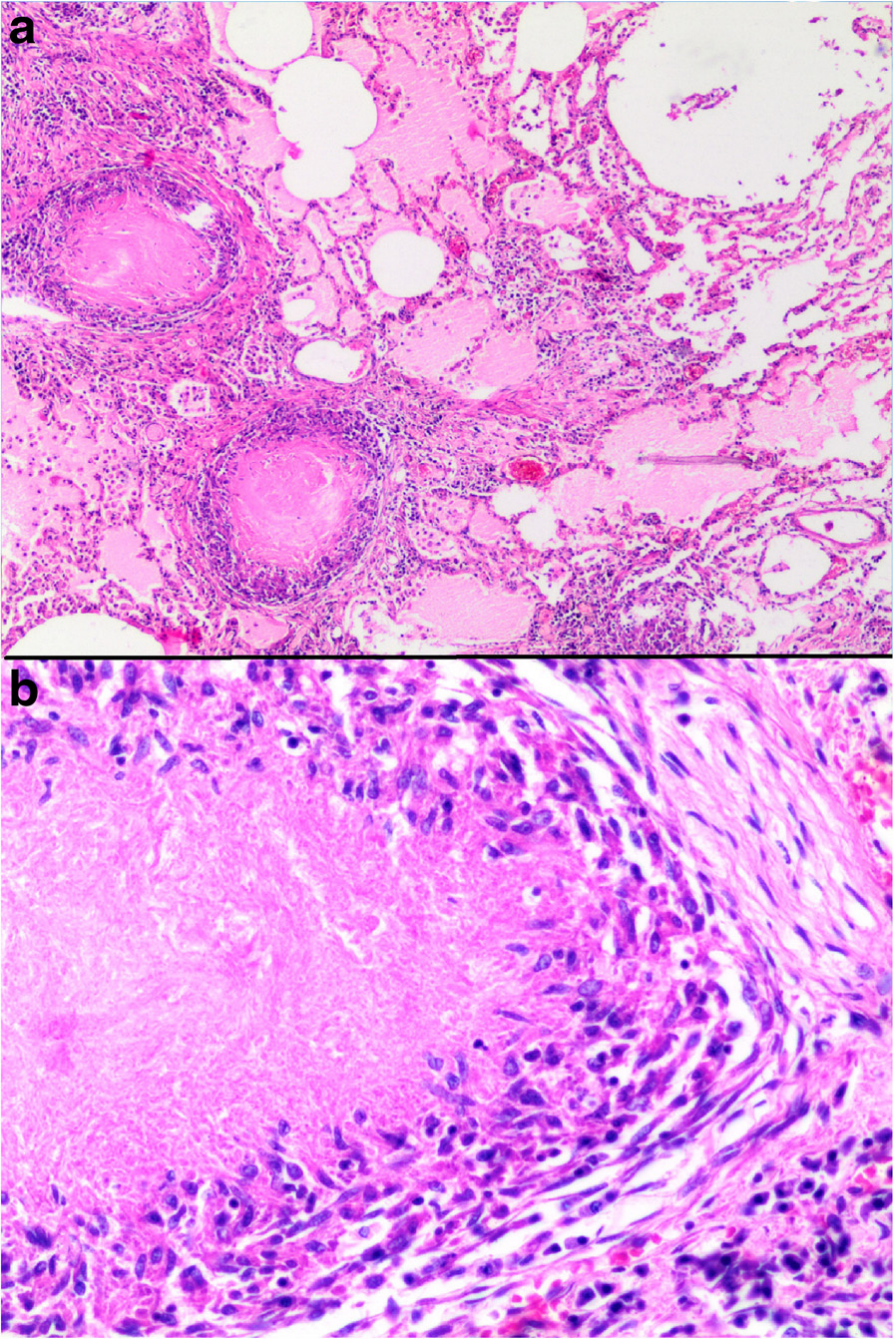
Microscopic view of tissues affected with tuberculosis in the dog, hematoxylin-eosin staining: **a**) two small granulomas with central area of necrosis in lung tissue–note proteinaceous fluid in surrounding alveoli; magnification 40×; **b**) details of granuloma in myocardium, note presence of central area of necrosis surrounded by epithelioid macrophages, connective tissue more peripherally and plasma cells infiltration (at the bottom right); magnification 200 ×

**Fig. 4:**
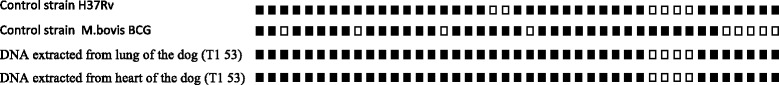
Spoligotype patterns of *Mycobacterium tuberculosis* isolates

Histopathological examination revealed numerous small granulomas in all grossly changed organs (Fig. 3a) consisting of central area of necrosis surrounded by a rim of macrophages and epithelioid cells, and peripheral proliferation of fibrous connective tissue (Fig. 3b). Langhans giant cells were not visible. In the slides stained with Ziehl-Neelsen stain, numerous acid-fast rods were observed, mostly in the cytoplasm of macrophages. As the macroscopic and microscopic findings suggested mycobacterial infection, lung and heart specimens were further investigated at the Institute of Tuberculosis and Lung Diseases in Warsaw. After decontamination with NALC-NaOH solution this material was inoculated onto Löwenstein-Jensen (L-J) slants and into liquid media Bactec Mycobacteria Growth Indicator Tube 960 (MGIT 960) (Becton Dickinson). Growth on liquid media was observed after 6 days (lung) and 8 days (heart). Then bacterial growth became visible also on the solid media. The presence of *Mycobacterium tuberculosis* complex (MTC) in our specimens was further confirmed with ET Direct Detection Assay. The BD ProbeTec ET was positive for *M. tuberculosis* complex DNA in both specimens. Both isolates were spoligotyped as described earlier [11]. Spoligotypes in binary format were converted to an octal code for comparison with the SITVIT2 proprietary database of the Pasteur Institute of Guadeloupe, which is an updated version of the previously released SpolDB4 database (http://www.pasteur-guadeloupe.fr:8081/SITVITDemo 30.05.2016). The spoligotype patterns obtained from both samples were identical and were represented by T1 53 pattern (Fig. 4).

Each isolate was tested for susceptibility to four first-line anti-tuberculosis agents: isoniazid (0.2 and 0.4 μg/ml), rifampicin (40 and 80 μg/ml), streptomycin (4 and 8 μg/ml) and ethambutol (2 and 4 μg/ml) using Method of Proportion (MOP) on L-J medium [12]. They all proved susceptible to all tested medicines.

The dog’s owners were educated about the potential health risk and asked to contact their physician. The chest radiographs excluded presence of active *M. tuberculosis* infection in the owner, his wife and their child.

## Discussion

Confirmation of a TB suspicion in a dog is complicated. The reaction to intradermally administered tuberculin is considered inconsistent and unreliable in this species [2]. Similarly, the result of the time-consuming repeated body temperature measuring in a suspected dog after subcutaneous tuberculin injection may be influenced by excitation or other factors. Also serologic testing gives in dogs unreliable results. In contrast, bacterial isolation is a commonly accepted reference standard method for the TB diagnosis both in humans and dogs [2] and was used in this study. The diagnosis was further confirmed by the BD ProbeTec ET Direct Detection Assay. This technique is based on amplification of MTC-specific IS6110 target DNA of 95 bp and detection by fluorescent-labeled probes. It is commercially available and allows the detection of MTC strains directly in clinical material. Spoligotyping used for genotyping of our isolates is a PCR-based method allowing to analyze strain-dependent polymorphisms observed in spacer sequences present within the direct repeat (DR) genomic region of MTC bacteria [11]. This locus contains multiple direct repeats, each of 36 bp, interspersed with non-repetitive, unique spacer sequences (spacers) of 35–41 bp in length. The number of direct repeats as well as the presence or absence of specific spacers reflect the polymorphic structure of the DR region and thus demonstrate variations between the strains [13]. This technique not only allows discrimination between *M. tuberculosis* and *M. bovis* (Fig. 4) but is also useful for classifying *M. tuberculosis* strains into spoligotype families and subfamilies [13]. In addition, it provides some important advantages over other genotyping techniques: simplicity, rapidity, high reproducibility and stability of the results, with the latter being expressed in a simple digital pattern, readily named and databased [14]. The spoligotype T1 53 found in our patient is the most common molecular *M. tuberculosis* pattern in humans in Poland. In a recent study in eastern Poland, this spoligotype represented as much as 36.4 % of total isolates [15]. This spoligotype is widespread worldwide with an increasing prevalence in Europe [15].

Certainly, our patient has suffered from *M. tuberculosis* infection for over one year as the tuberculoma was first seen in echocardiography almost 12 months before euthanasia. Nevertheless, TB in companion animals is regarded as such a rare condition in Poland that it is very likely to remain misdiagnosed or undetected [9, 10]. This happened also in our patient in which TB remained undiagnosed ante mortem despite several visits to different veterinarians over a period of virtually one year. Even though circulatory and respiratory symptoms periodically aggravated, and the general condition of the dog tended to deteriorate, TB was not even taken into consideration. This case shows that veterinarians should be alert and consider TB when confronted with chronically ill emaciating patients also in countries where TB is under control both in humans and cattle.

Lack of proper ante mortem diagnosis could be partially attributed to an atypical location of the main tuberculous lesions. Typically, TB in dogs affects lungs and regional lymph nodes, however, lesions have been described also in other organs including the central nervous system, spleen, omentum, pancreas, liver, kidneys, testis, pericardium and diaphragm [9, 10, 16–21]. So far the only heart abnormalities described in canine TB were grayish-white, circumscribed lesions in the pericarium and serous pericardial fluid [9]. The patient in this report had a nodule near the left atrium of the heart seen in echocardiography, and post mortem examination revealed tuberculomas in both atria. Typically, multifocal nodular lesions predominate in canine TB [2, 16–21]. An intracranial tuberculoma has been reported recently in a dog [16]. To our best knowledge, this is the first report of the heart tuberculoma in a dog.

Even in humans tuberculomas are a rare manifestation of TB, mostly seen in the central nervous system [22]. In the past, cardiac tuberculomas in humans had been occasionally described only as post mortem findings. Nowadays, along with advances of imaging diagnostics, they can be observed as single or multiple lesions of different location and size [22–25]. The diagnosis is usually based on biopsy, as non-invasive methods yield inconclusive results. In humans, pericardial fluid in the course of TB is also yellow, like it was in our patient, and *M. tuberculosis* DNA can be detected by PCR [25]. TB in humans can result in the thickened and calcified pericardium, often referred to as “porcelain heart” [26]. Similar autopsy finding was seen in our patient.

The source of infection in our patient remains unknown. None of the family members in this household seemed to be infected with *M. tuberculosis*. The dog was bought to this household as a 4-month-old puppy and spent its whole life with this family. They lived in a single-family detached home in a suburban area and the dog had access to the garden. No contacts with a person known to suffer from TB were revealed in dog’s medical history. *M. tuberculosis* infections in dogs and cats are believed to be transmitted from people to animals through close direct contacts [2]. Airborne transmission is common, however alimentary infections are also possible, when a dog is exposed to contaminated food. Disseminated *M. tuberculosis* infection has been recently described in a dog that was on several occasions observed licking the expectorated secretions of his owner suffering from tuberculosis [9]. In experimental conditions infected dogs may transmit *M. tuberculosis* to other dogs by close contact [27]. As our patient used to spend a lot of time in the garden surrounded by a wire mesh netting fence, some nose-to-nose contacts with other animals (e.g. mutual barking up) or even feeding with leftovers by foreign people could not be excluded. In our case, the owners remained uninfected despite they had been living in close contact with the infected dog for at least one year. Likewise, Posthaus et al. reported that a dog with disseminated *M. tuberculosis* TB did not transmit the infection to the owners [28]. These observations indicate that dog-to-human transmission of *M. tuberculosis* seems to be an uncommon situation [2].

## Conclusion

Companion animals may occasionally suffer from tuberculosis. Typically, TB affects lungs and regional lymph nodes but lesions have been described also in other organs. Majority of cases probably remain misdiagnosed or undetected. Even in humans tuberculomas in a heart are a rare manifestation of *Mycobacterium* infection diagnosed mostly post mortem. Lump in the area of the heart diagnosed during echocardiography examination, particularly if accompanied by respiratory symptoms should make veterinarians include mycobacterial infection in the list of differential diagnoses.

## Abbreviation

Ao, aorta; DR, direct repeat; EF, ejection fraction; FS, fractional shortening; IVSd, interventricular septum at end diastole; IVSs, interventricular septum at end systole; LA, left atrium; LA/Ao, left atrium: aortic root ratio; L-J, Löwenstein-Jensen; LVIDd, left ventricular internal diameter at end diastole; LVIDs, left ventricular internal diameter at end systole; LVOT, left ventricular outflow tract; LVPWd, left ventricular free wall thickness at end diastole; LVPWs, left ventricular posterior wall thickness at end systole; MTC, *Mycobacterium tuberculosis* complex; RV, right ventricle; RVOT, right ventricular outflow tract; TB, tuberculosis
